# Epidemiological Characteristics of Human Rabies in Chongqing, China, 2016–2024

**DOI:** 10.3390/tropicalmed11010030

**Published:** 2026-01-22

**Authors:** Longyu Chen, Yi Yuan, Yu Xia, Jiang Long, Zhijin Li, Tingting Li, Li Qi

**Affiliations:** 1 Chongqing Municipal Center for Disease Control and Prevention (Chongqing Academy of Preventive Medicine), Chongqing 400707, China; chenlongyu24@163.com (L.C.); cqcdcyy@163.com (Y.Y.); xiayucq4@hotmail.com (Y.X.); 68803648@163.com (J.L.); lzj514@foxmail.com (Z.L.); lee712ting@126.com (T.L.); 2 School of Public Health, Southwest Medical University, Luzhou 646000, China

**Keywords:** human rabies, epidemiological characteristics, post-exposure prophylaxis

## Abstract

(1) Background: Human rabies continues to be a significant public health challenge and imposes a heavy disease burden. The epidemiological characteristics and post-exposure prophylaxis (PEP) of human rabies in Chongqing were analyzed to provide a scientific basis for its prevention and control in Chongqing. (2) Methods: Data and case investigation forms of the human rabies epidemic in Chongqing from 2016 to 2024 were collected and analyzed using descriptive epidemiological methods. (3) Results: From 2016 to 2024, 84 human rabies cases were reported in Chongqing, with an average annual incidence rate of 0.03 per 100,000 population. Among the cases, 72.6% were aged 45 and above. Farmers constituted the primary infected group (73.8%). Analysis of exposure patterns and PEP revealed that 92.4% of cases involved dog transmission, with domestic dogs responsible for 65.2% and stray dogs for 31.8%. After exposure, 51.5% received no treatment, while only 6 individuals were vaccinated against rabies. (4) Conclusions: Although rabies incidence in Chongqing is low, dogs remain the primary source, and post-exposure vaccination is often delayed. Strengthening health education and dog immunization is crucial for supporting the global “Zero by 30” target.

## 1. Introduction

Rabies is a zoonotic encephalitis caused by the highly neurotropic rabies virus that affects all mammals, including humans. Following transmission via bites, scratches, or mucosal contact (such as licking of open wounds or the conjunctiva) with infectious animal saliva, the virus ascends through neural pathways to the central nervous system [1,2]. Once it invades the central nervous system and clinical symptoms appear, the disease typically progresses rapidly and is fatal within about 10 days, with a mortality rate approaching 100%. A prolonged and variable incubation period, which can last from months to years, precedes symptom onset [3,4]. Therefore, timely and standard post-exposure prophylaxis (PEP) is crucial for blocking the transmission of rabies. Approximately 99% of human rabies cases are caused by dogs, especially in developing countries with low canine immunization rates (Asia and Africa). However, in areas that have successfully eliminated dog-transmitted rabies, wildlife—including bats, ferret badgers, foxes, mongooses, raccoons, raccoon dogs, and skunks—become the key reservoir hosts for the rabies virus [5].

Currently, rabies is prevalent in more than 150 countries and regions globally, with Asia and Africa being the most affected. It leads to about 59,000 deaths each year. Notably, 40% of the deceased are children under the age of 15, rendering rabies the leading cause of death among zoonotic diseases [4]. Despite the availability of effective vaccines for both humans and animals, rabies still results in the loss of approximately 3.7 million disability-adjusted life years (DALYs) and an economic burden of $8.6 billion each year [6]. In order to control and eliminate rabies, the World Health Organization, the World Organisation for Animal Health, the Food and Agriculture Organization of the United Nations, and the Global Alliance for Rabies Control jointly launched the global strategic initiative “Zero by 30” in 2015, with the goal of eliminating human deaths from dog-mediated rabies by 2030 through multi-pronged interventions [7].

In China, rabies outbreaks have experienced three significant fluctuations (1950–1960, 1960–1995, and 1995–2023), with annual cases once reaching over 7000, making it a critical public health issue [8]. In recent years, China has actively responded to international initiatives and implemented multiple prevention and control measures, the incidence of human rabies has continued to decline [9,10]. The year 2007 marked the peak of the recent epidemic, with 3300 reported cases across 984 counties and districts. By 2023, the number of cases had dropped by 96.3% compared to 2007, with only 122 cases reported nationwide and an 89.7% reduction in the number of affected counties and districts (down to 101). Currently, several provincial-level administrative regions in China have reported zero or single-digit cases, indicating a clear trend of sporadic occurrence.

However, rabies still poses a significant disease burden in China [11]. Chongqing is the largest municipality directly under the Central Government of China and is located in an area with a high incidence of human rabies (such as Hunan Province and Sichuan Province), posing a significant risk of disease transmission. To elucidate the epidemiological patterns of human rabies in Chongqing, we conducted an in-depth epidemiological analysis by examining the surveillance data and individual case records from 2016 to 2024. This study aimed to provide a robust epidemiological basis for effective control and prevention of rabies in this area.

## 2. Materials and Methods

### 2.1. Ethics Statement

The data for this study were obtained from the ongoing public health surveillance of a statutorily notifiable infectious disease. In accordance with regulations issued by China’s National Health Commission, the investigation of human rabies cases constitutes a routine public health monitoring activity and is therefore exempt from review by an institutional ethics committee. All data analyzed were de-identified and contained no personal identifiable information. Every investigation was carried out following the provision of verbal informed consent by the patients’ family members.

### 2.2. Data Collection

Incidence data for human rabies in Chongqing from January 2016 to December 2024 were sourced from China’s National Notifiable Disease Reporting System (NNDRS). According to the law of the People’s Republic of China on Prevention and Treatment of Infectious Diseases, all human rabies cases should be reported to NNDRS within 24 h after diagnosis. This system serves as a nationwide unified online direct reporting platform for notifiable infectious diseases, covering medical institutions at all levels, which ensures the timeliness and completeness of data.

To investigate the situation of rabies exposure and the PEP of human rabies cases, trained investigators from local Centers for Disease Control and Prevention (CDCs) conducted face-to-face interviews with 66 cases or their family members using a standardized questionnaire, after obtaining informed consent. The questionnaire covered four parts: (a) the cases’ demographic profile (name, gender, age, occupation); (b) exposure characteristics (date of event, site of lesion, the animal vector, the category of exposure); (c) PEP (wound washing, vaccination and/or immunoglobulin administration); and (d) clinical manifestation.

The yearly population statistics for Chongqing were derived from the official Statistical Yearbook of the Chongqing Municipal Bureau of Statistics.

### 2.3. Study Area

**Figure 1:**
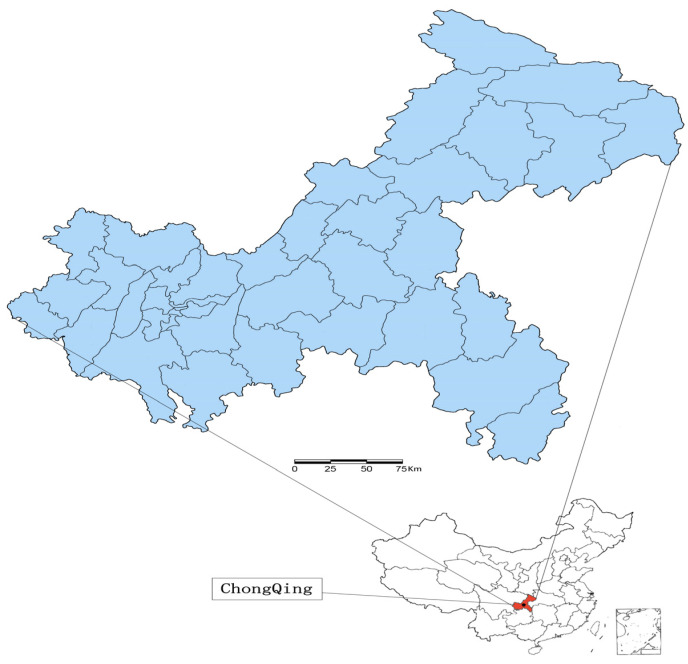
Geolocation of Chongqing in China.

Chongqing is one of China’s four direct-administered municipalities. It is geographically located in southwestern China (28°10′–32°13′ N, 105°11′–110°11′ E) and has a land area of 82,400 square kilometers, as shown in Figure 1. The municipality is home to a population of over 34 million residents.

### 2.4. Diagnostic Criteria for Rabies

Rabies is classified into two types: the furious type and the paralytic type. The furious type, accounting for approximately 80% of cases, is characterized by agitation, aggression, hydrophobia, and aerophobia. In contrast, the paralytic type presents with progressive flaccid paralysis, often starting from the wound site, accompanied by sensory impairment. Both forms are progressive and inevitably fatal following the onset of clinical symptoms. Additionally, atypical manifestations have been reported, particularly in cases associated with bats. According to the Rabies Diagnosis and Treatment Guidelines (2021) [12], rabies cases are categorized into clinically diagnosed and laboratory-confirmed types.

Clinical diagnosis:

(1) A clear epidemiological history, namely a history of being bitten, scratched, or having mucous membranes licked by a suspected animal such as a dog or cat prior to the onset of illness;
(2) Typical clinical manifestations, such as hydrophobia, aerophobia, pharyngeal muscle spasms, progressive paralysis, and other rabies-specific symptoms;
(3) The preliminary exclusion of other known etiologies.

A case can be clinically diagnosed if there is an epidemiological history and the clinical manifestations are consistent with rabies.

Laboratory diagnosis: On the basis of clinical diagnosis, any one of the following laboratory test results confirms the diagnosis of a confirmed case:

(1) Positive isolation of the rabies virus;
(2) Positive detection of rabies virus antigen by the fluorescent antibody test (FAT), direct rapid immunohistochemical test (dRIT), or enzyme-linked immunosorbent assay (ELISA);
(3) Positive detection of rabies virus nucleic acid;
(4) Positive detection of rabies virus-neutralizing antibodies, as determined by the mouse neutralization test (MNT) or the rapid fluorescent focus inhibition test (RFFIT), in an individual without a history of rabies vaccination.

### 2.5. Exposure Category

Rabies exposure is classified into three levels according to the type of exposure and degree of exposure: Category I is touching or feeding animals, or animal licks on intact skin (no exposure). Category II is nibbling of uncovered skin, minor scratches, or abrasions without bleeding (exposure). Category III is single or multiple transdermal bites or scratches, contamination of mucus membrane or broken skin with saliva from animal licks, or exposures due to direct contact with bats (severe exposure) [13].

### 2.6. Statistical Analysis

Statistical analysis was performed using Excel 2019 (Microsoft Corporation, Redmond, WA, USA). Descriptive epidemiological methods were applied, with incidence rates, proportions, and medians used to summarize the demographic characteristics of the cases. The Chi-square test and the Kruskal–Wallis rank-sum test were employed to compare incubation periods across groups with different exposure characteristics. A *p*-value of less than 0.05 was considered statistically significant.

## 3. Results

### 3.1. Epidemiological Characteristics

**Figure 2:**
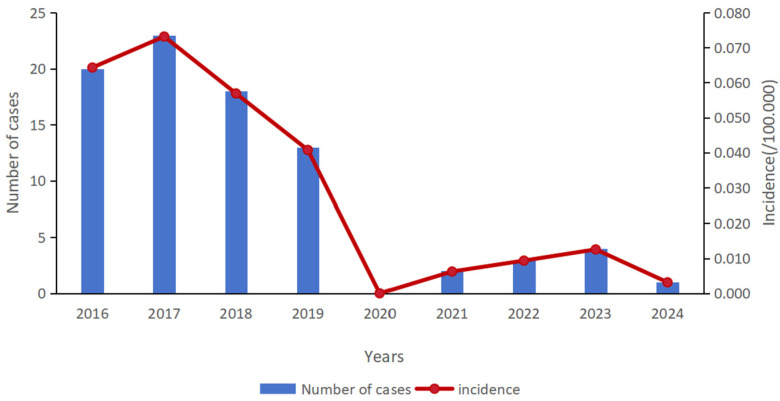
The number of human rabies cases and the incidence rate in Chongqing, China, 2016–2024.

From 2016 to 2024, 84 rabies cases were reported in Chongqing. The average annual incidence rate was 0.03 cases per 100,000 population. From 2016 to 2024, the incidence of human rabies showed a declining trend, reaching its lowest point in 2020 (with fewer than 5 cases from 2020 to 2024) (Figure 2).

**Figure 3:**
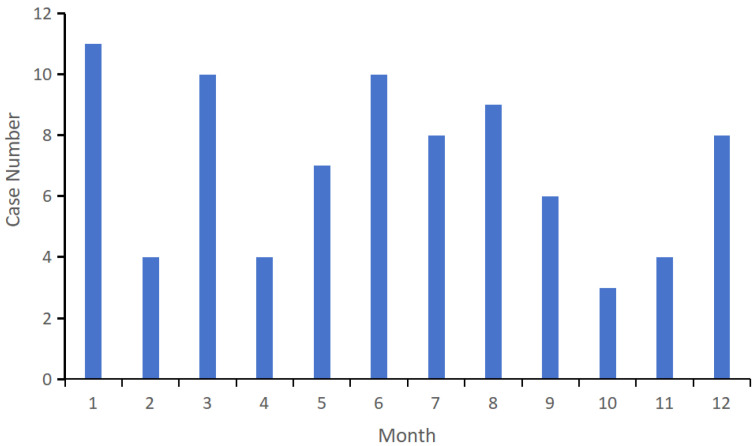
The human rabies cases by month in Chongqing, China, 2016–2024.

During the study period, no cases occurred in some months each year, and no cases were reported throughout 2020. Grouped by season, summer (June to August) recorded the highest number of cases, with 27 cases, accounting for 32.1% of the total. This was followed by winter (December to February) with 23 cases (27.4%), spring (March to May) with 21 cases (25.0%), and autumn (June to August) with 13 cases (15.5%) (Figure 3). There was no obvious seasonal pattern in the incidence of the disease.

Among the 84 cases, 56 were male and 28 were female, with a male-to-female ratio of 2:1. The age range of onset was from 4 to 90 years, with a median age of 60. The highest proportion (52.4%) fell within the ≥60-year-old group, followed by the 45–59-year-old group at 20.2%. Farmers constituted the most common occupational group (62 cases, 73.8%), while students and children (9 cases, 10.7%) were the second most common. These two groups collectively accounted for over 80% of the total affected population (Table 1). There were 25 districts and counties in Chongqing with reported rabies cases. Dazu District reported the highest number of cases (14 cases, 15.9%), followed by Hechuan District (13 cases, 14.8%). Other regions and counties had fewer cases (less than 5 cases), with scattered distribution.

### 3.2. Exposure and Incubation

A total of 66 rabies case investigation forms were collected. Among the exposure methods for rabies, there were 55 cases (83.3%) of direct bite, 8 cases (12.1%) of scratching, and 3 cases (4.5%) of other exposure methods. Among these, 47 (71.2%) were classified as Grade III exposure, 18 (27.3%) as Grade II, and 1 (1.5%) as Grade I. The most common exposure sites were upper limbs (43 cases, 65.2%) and lower limbs (23 cases, 34.8%) (Table 2). Of the 66 cases, 46 had clear exposure times, with a median incubation period of 69.5 days, with the shortest being 11 days and the longest being 5 years. For cases with clearly documented exposure dates, the incubation period was compared by exposure route, exposure degree, and exposure site. The results showed no statistically significant differences in the incubation period across different exposure routes or degrees (*p* > 0.05). However, the incubation period for head and neck exposures, with a median of 14 days, was significantly shorter than that for upper and lower limb exposures (*p* < 0.05) (Table 3).

Most patients exhibited typical symptoms of rabies, including agitation (54, 81.9%), hydrophobia (49, 74.2%), Anemophobia (45, 68.2%), and photophobia (34, 51.5%), convulsions (32, 48.5%), and mental disorders (33, 50.0%). In 60 cases (90.9%), the combination of two or more of the aforementioned symptoms was observed. Standardized management is key to preventing rabies, including thorough wound cleansing and disinfection after exposure, along with vaccination and administration of passive immunizing agents based on exposure categorization. Regarding post-exposure management, 34 patients (51.5%) did not receive wound treatment, 28 (42.4%) treated themselves, and 4 (6.1%) sought medical care. Among the patients with category Ⅲ exposure, 6 received vaccination, of whom 3 also received immunoglobulin injections while the other 3 received vaccination only. All 6 of these patients eventually died. Among the fatal cases, 1 completed the full vaccination course, 4 died before completing the vaccination series, and 1 failed to complete the full course due to financial constraints.

Of the animals that hurt people, 61 (92.4%) were dogs and 5 (7.6%) were cats. These animals were primarily domesticated pets (including pets kept by the owner and neighbors) (43 cases, 65.2%) or stray animals (21 cases, 31.8%). Regarding the causes of animal bites, 36 cases (52.2%) were due to intentional attacks, 13 (19.4%) occurred during playful interactions, and 10 (14.9%) resulted from self-defense (Table 2).

## 4. Discussion

Through actions such as increasing the coverage of standardized rabies prevention clinics, launching large-scale dog vaccination programs, enforcing mandatory registration, capturing stray dogs, strengthening surveillance, establishing a multi-departmental coordination mechanism (involving agriculture, health, public security, and urban management authorities, etc.), and conducting sustained public education campaigns, Chongqing has effectively controlled rabies transmission and maintained low prevalence. From 2016 to 2024, the number of reported human rabies cases in Chongqing continuously decreased, reaching its lowest point in 2020 (with fewer than 5 cases reported from 2020 to 2024). The incidence of rabies in Chongqing is lower than the overall incidence level in China, and it is sporadic with low risk of disease [14,15,16]. It is noteworthy that between 2020 and 2022, China implemented widespread non-pharmaceutical interventions, such as restrictions on human mobility and reduced gatherings, to control the COVID-19 pandemic. These measures likely significantly reduced the frequency of contact between humans and dogs, particularly stray dogs, thereby substantially lowering the risk of rabies exposure in the short term. This may have been an important factor contributing to the lower incidence of rabies during the 2020–2022 period.

The primary target groups for this study include males, farmers, and individuals aged 45 and above, consistent with previous studies [9,15]. Individuals in these groups face a higher risk of developing the disease due to occupational, behavioral, and other factors. Research indicates that males are more likely to mishandle wounds or remain passive after rabies exposure [17]. In rural areas, frequent stray dog activity increases farmers’ vulnerability to dog attacks during fieldwork. Additionally, lower dog vaccination coverage in these regions elevates the risk of viral transmission among dogs. Coupled with limited accessibility to PEP clinics and insufficient public awareness of rabies, these factors collectively heighten the infection risk for this population [18,19,20,21,22]. Therefore, expanding the geographic coverage and service capacity ofPEP clinics is essential. In parallel, raising public awareness about the need for timely preventive measures is crucial to achieving effective rabies control.

While approximately 40% of rabies cases in many low- and middle-income countries of Africa and Asia occur in children under 15 years, this age group constituted only 10.7% of cases in our study [4]. This marked difference may be closely related to the sustained and targeted health education carried out locally [8,23]. Through school- and community-based awareness campaigns, both children and their families have significantly improved their understanding of rabies risks, thereby reducing unprotected contact with non-immunized dogs and lowering exposure risk in this age group.

The median incubation period for rabies is 69.5 days, which is consistent with the incubation period (1–3 months) stipulated by the WHO [4]. Rabies is a preventable but incurable disease. If an exposed person is treated in a standardized and timely manner after exposure, the risk of exposure can be greatly reduced [24]. This study observed that the limbs were the primary sites of rabies exposure. However, analysis of disease progression across different bite locations revealed that patients with bites to the head or neck experienced significantly faster clinical deterioration compared to those with limb bites, a finding consistent with other studies [25]. This is primarily attributed to the dense neural innervation and close anatomical proximity of the head and neck region to the central nervous system. In the absence of timely and appropriate intervention, the virus can rapidly invade along neural pathways, leading to a substantially elevated risk of disease progression [26].

In post-exposure management, 51.5% of patients did not receive wound treatment after exposure, and among those who received treatment, the rates of wound treatment, rinsing, disinfection, suturing, and vaccination were low. Only six patients received the rabies vaccine, three of whom also received immunoglobulin. Issues such as delayed exposure treatment, incomplete preventive measures and insufficient emergency vaccinations still persist. In order to prevent the occurrence of rabies, it is necessary to continuously strengthen public health education, with a focus on improving awareness among high-risk groups regarding timely and standardized post-exposure management, in order to reduce delays caused by insufficient knowledge. At the same time, financial support and service accessibility should be improved to alleviate the economic burden on patients and ensure that all exposed individuals can access complete and standardized preventive treatment without barriers. Furthermore, efforts should be made to enhance the capacity of healthcare institutions by strengthening standardized training for primary healthcare workers and implementing rigorous quality supervision of post-exposure management procedures, thereby ensuring the timeliness and standardization of wound treatment, vaccination, and the use of immunoglobulins.

Rabies exposure is mainly caused by dog exposure, which imposes a great economic burden [6,27]. It is estimated that there are 80 million to 200 million dogs in China, which serve as the primary reservoir host for the rabies virus [28]. Approximately 95% of human cases are caused by dog-mediated transmission. In the central, eastern, and southern regions of the country, dogs remain the most significant vector of the disease and the leading source of human bite exposures [29]. Domestic and stray dogs remain the primary sources of rabies transmission in Chongqing, which is consistent with previous research findings in the city [30]. However, their numbers have now significantly declined. The Chongqing Canine Management Regulations [31] provide clear guidelines for the management of domestic and stray dogs, effectively controlling the risk of transmission from animal sources.

To achieve the 2030 goal of eliminating rabies, maintaining a dog vaccination coverage rate above 70% is essential [32]. In these cases, most of the vaccination status of domestic dogs was not filled in, and the number of rabies cases in recent years is at a low level, so it is difficult to accurately assess the immunization status of dogs. However, according to a government report [33], Chongqing Municipality cumulatively completed 558,700 dog doses of rabies vaccination in 2024, with a vaccination density of 99.62% and a positive rate of immune antibodies of 73.11%. Moving forward, strengthening dog registration, vaccination programs, and stray management will reinforce immunization barriers and provide sustained support for regional rabies control.

In recent years, alongside significant shifts in pet ownership patterns and a rapid increase in the domestic cat population, cats have emerged as an important and increasingly prominent source of rabies exposure [34]. Surveillance data from multiple cities indicate a consistent upward trend in the proportion of exposures caused by cats during post-exposure prophylaxis management. For instance, monitoring in Beijing shows that since 2020, the proportion of exposures caused by cat injuries has surpassed that caused by dogs, becoming the primary source of exposure [35]. Similarly, a study on rabies vaccination compliance in Shenzhen also notes that the number of patients seeking treatment for cat-related injuries far exceeds those injured by dogs [36]. It is noteworthy that cat scratches are more common and easily overlooked, and coupled with their relatively concealed activity patterns, this often leads to delays in post-exposure management. Therefore, within current rabies prevention and control efforts, there is an urgent need to enhance focus on cat-related exposures and to integrate cats into an immunization and registration management system of equal importance as that for dogs, in order to establish a more comprehensive barrier against animal-borne diseases.

This study has several limitations. First, most cases were clinically diagnosed without conclusive laboratory confirmation, which may have led to some degree of misdiagnosis. Second, during case investigations, information about some patients with long incubation periods or in coma mainly came from family members. This not only may result in incomplete or biased PEP records but also may cause recall bias.

## 5. Conclusions

In conclusion, the incidence of rabies in Chongqing has been consistently maintained at a low level from 2016 to 2024. To achieve the established goal of eliminating rabies by 2030, it is essential to intensify efforts in key areas. These include conducting targeted health education for high-risk groups, expanding the coverage of large-scale dog vaccination programs, and systematically strengthening the surveillance system for the rabies pathogen.

## Figures and Tables

**Table 1:** Characteristics of human rabies cases in Chongqing, China, 2016–2024.

	Number of Human Rabies Cases	Proportion (%)
Age group		
0–14 years	9	10.7
15–29 years	5	6.0
30–44 years	9	10.7
45–59 years	17	20.2
≥60 years	44	52.4
Gender		
Male	56	66.7
Female	28	33.3
Occupation		
Farmer	62	73.8
Students and children	9	10.7
Housewife or unemployment	5	6.0
Worker	2	2.4
Others	6	7.1

**Table 2:** Exposure characteristics of human rabies cases in Chongqing, China, 2016–2024.

	No. of Human Rabies Cases	Proportion (%)
Exposure method		
Bite	55	83.3
Scratch	8	12.1
Others	3	4.5
Exposure level		
I	1	1.5
II	18	27.3
III	47	71.2
Exposure site		
Head, neck and Trunk	6	9.1
Upper limb	43	65.2
Lower limb	23	34.8
Exposure of multiple parts	3	4.5
Wound treatment method		
Unprocessed	34	51.5
Handle it by yourself	28	42.4
Medical institution handling	4	6.1
Animal vector		
Dog	61	92.4
Cat	5	7.6
Animal source		
Domesticated animal	43	65.2
Stray animals	21	31.8
Wild animals	0	0.0
Other	2	3.0
Exposure reasons		
Active attack by the vector	35	52.2
Defensive attack by the vector	10	14.9
Playing with the vector	13	19.4
Other	6	9.0

**Table 3:** Analysis of incubation period of rabies in different exposure mode, degree and location in Chongqing, China, 2016–2024.

	Median (Interquartile Range) (Days)	*p* Value
Exposure method		>0.05
Bite	69 (41.75, 135.25)	
Scratch	151.5 (83.5, 229.25)	
Others	38.5 (28.75, 48.25)	
Exposure level		>0.05
II	120 (69, 272)	
III	62 (41.5, 125)	
Exposure site		<0.05
Upper limb	63 (41.5, 125)	
Lower limb	120 (64.5, 263)	
Head, neck and Trunk	14 (13.2, 22)	

## Data Availability

The datasets used and/or analyzed during the current study are not publicly available to protect the privacy and confidentiality of the study participants. However, they are available from the corresponding author upon reasonable request. Requests will be evaluated based on a sound scientific proposal and require a completed data sharing agreement, contingent on approval by the original ethics committee.

## Abbreviations

The following abbreviations are used in this manuscript:

PEP	Post-exposure prophylaxis
RIG	Immunoglobulin
WHO	World Health Organization
NNDRS	China’s National Notifiable Disease Reporting System
